# Alternative radiopacifiers for polymethyl methacrylate bone cements: Silane-treated anatase titanium dioxide and yttria-stabilised zirconium dioxide

**DOI:** 10.1177/0885328220983797

**Published:** 2021-02-11

**Authors:** Wayne Nishio Ayre, Nicole Scully, Carole Elford, Bronwen AJ Evans, Wendy Rowe, Jeff Rowlands, Ravi Mitha, Paul Malpas, Panagiota Manti, Cathy Holt, Rhidian Morgan-Jones, James C Birchall, Stephen P Denyer, Sam L Evans

**Affiliations:** 1 School of Dentistry, Cardiff University, Cardiff, UK; 2 School of Medicine, Cardiff University, Cardiff, UK; 3 School of Engineering, Cardiff University, Cardiff, UK; 4 School of Environment, Ionian University, Zakynthos, Greece; 5 Department of Trauma & Orthopaedics, Cardiff & Vale University Health Board, Cardiff, UK; 6 School of Pharmacy and Pharmaceutical Sciences, Cardiff University, Cardiff, UK; 7 School of Pharmacy and Biomolecular Sciences, University of Brighton, Brighton, UK

**Keywords:** PMMA, bone cement, radiopacifier, silane, mechanical, biocompatibility, toxicity

## Abstract

Poly (methyl methacrylate) (PMMA) bone cement is widely used for anchoring joint arthroplasties. In cement brands approved for these procedures, micron-sized particles (usually barium sulphate, BaSO_4_) act as the radiopacifier. It has been postulated that these particles act as sites for crack initiation and subsequently cement fatigue. This study investigated whether alternative radiopacifiers, anatase titanium dioxide (TiO_2_) and yttria-stabilised zirconium dioxide (ZrO_2_), could improve the *in vitro* mechanical, fatigue crack propagation and biological properties of polymethyl methacrylate (PMMA) bone cement and whether their coating with a silane could further enhance cement performance. Cement samples containing 0, 5, 10, 15, 20 and 25%w/w TiO_2_ or ZrO_2_ and 10%w/w silane-treated TiO_2_ or ZrO_2_ were prepared and characterised *in vitro* in terms of radiopacity, compressive and bending strength, bending modulus, fatigue crack propagation, hydroxyapatite forming ability and MC3T3-E1 cell attachment and viability. Cement samples with greater than 10%w/w TiO_2_ and ZrO_2_ had a similar radiopacity to the control 10%w/w BaSO_4_ cement and commercial products. The addition of TiO_2_ and ZrO_2_ to bone cement reduced the bending strength and fracture toughness and increased fatigue crack propagation due to the formation of agglomerations and voids. Silane treating TiO_2_ reversed this effect, enhancing the dispersion and adhesion of particles to the PMMA matrix and resulted in improved mechanical properties and fatigue crack propagation resistance. Silane-treated TiO_2_ cements had increased nucleation of hydroxyapatite and MC3T3-E1 cell attachment *in vitro*, without significantly compromising cell viability. This research has demonstrated that 10%w/w silane-treated anatase TiO_2_ is a promising alternative radiopacifier for PMMA bone cement offering additional benefits over conventional BaSO_4_ radiopacifiers.

## Introduction

Polymethyl methacrylate (PMMA) bone cement was first introduced into orthopaedic surgery in the late 1950s when Sir John Charnley used the material to successfully anchor a hip replacement to bone.^1^ This procedure has since grown in popularity, with recent data from the National Joint Registry (for England, Wales, Northern Ireland, the Isle of Man and the States of Guernsey) indicating in 2018, approximately 28% of primary hip procedures and 86% of primary knee procedures used PMMA bone cement.^2^ PMMA has also shown promise for other biomedical applications, such as tissue engineering,^3–5^ and as a vehicle for delivering a wide range of therapeutics.^6–9^ Although joint replacements are considered to be one of the most successful surgeries of the 20^th^ century, data from the National Joint Registry has shown that approximately 14,920 revision surgeries were performed in 2018 for failed hip and knee replacements (7% of all hip and knee procedures), with aseptic loosening being the major cause of failure, accounting for approximately 40% of revisions.^2^

As the probability of aseptic loosening in cemented joint replacements increases over time, cement fatigue is likely involved in the failure process.^2,10^ Although the shape and surface texture of implants have been optimised to improve the distribution of stresses across the cement mantle,^11–13^ internal stress concentrations within the cement still arise from pores, voids and radiopacifier agglomerations.^14–20^ Retrieval studies have shown these stress concentrations to initiate fatigue cracks with subsequent cyclic loading and overloads causing these cracks to propagate and coalesce over prolonged periods of time.^21,22^ This gradually weakens the cement, resulting in fatigue failure below the maximum strength of the material.^23^

As PMMA does not effectively attenuate X-rays, radiopacifiers, such as micron-sized barium sulphate (BaSO_4_) particles, have been approved for commercial bone cements. The addition of inorganic particles (8–15%w/w of the powdered component) allows the location, penetration and integrity of the cement to be observed non-invasively using X-ray imaging. The effect of radiopacifiers on the mechanical and fatigue crack growth rates of PMMA bone cement has been widely researched. Baleani and Viceconti^24^ showed that adding 10%w/w BaSO_4_ to PMMA lowered the endurance limit and fracture toughness of the material by 13% but also decreased the crack growth rate by up to 66%. A study by Ginebra et al.^25^ found that BaSO_4_ decreased the tensile strength but improved the crack propagation resistance of PMMA. Lewis^10^ highlighted the positive effect radiopacifiers can have in reducing crack propagation by drawing on 6 different studies.^26–31^ It was hypothesised that inorganic filler particles delay crack propagation by redirecting and deviating the crack tip. Conversely, other studies have shown agglomerations of radiopacifiers to be detrimental to the fatigue life of the cement.^16–20^ These studies showed agglomerations to act as sites for crack initiation, with debonding of the radiopacifier from the PMMA matrix also creating defects in the material.^15–20^ An analytical study using stress analysis and fracture mechanics by Evans^32^ also found that large defects, such as those caused by agglomerations, can cause a disproportionate increase in crack growth rates. Therefore, although radiopacifiers have been shown to reduce crack propagation, large agglomerates are also thought to be a source for crack initiation and a disproportionate increase in crack growth.

An additional area of concern surrounding the radiopacifiers used in bone cements is the potential effect on osteolysis and bone resorption. *In vitro* studies have shown wear debris and radiopacifier particles from PMMA bone cement induced high levels of inflammatory cytokine production from monocytes and enhanced osteoclast differentiation.^33–35^ *In vivo* rat tibial implantation and subcutaneous pouch studies have confirmed similar findings.^34,36^ These studies have also shown BaSO_4_ radiopacifiers to be more detrimental than PMMA particles or ZrO_2_ radiopacifiers, inducing higher levels of inflammation and bone resorption.^35,36^ To overcome these issues, focus has been placed on the development of iodine-based radiopacifiers and nano-sized radiopacifiers;^37–39^ however concerns regarding allergic reactions to iodine and the potential systemic effects of nanoparticles have limited their clinical translation.^40,41^

The use of alternative micron-sized radiopacifiers with unique material properties may help overcome some of the issues previously outlined. Yttria-stabilised ZrO_2_ for example, has unique transformation toughening mechanisms. When heated above 950 °C, the crystal structure of ZrO_2_ converts to a tetragonal arrangement which is accompanied by a 1–4% volumetric reduction.^42^ Addition of stabilisers such as yttria helps maintain this crystal structure upon cooling. When a crack tip approaches the yttria-stabilised ZrO_2_, the tetragonal crystal structure reverts to a monoclinic phase and due to its increase in volume, applies compression to the crack tip, slowing crack growth.^42^ Other materials such as titanium dioxide (TiO_2_) may offer alternative benefits such as increased calcium ion interaction, which is important for protein and osteoblast attachment as well as hydroxyapatite (HA) formation.^43^ The anatase crystal structure of TiO_2_ is speculated to have a higher bioactivity than rutile or amorphous TiO_2_ due to the closer lattice match with HA, higher acidity and lower surface zeta potential caused by a larger number of surface hydroxyl groups.^44,45^ Deposition of HA on anatase at a pH of 7.4 has been shown to be faster than on the rutile form at the same pH.^44^

**Figure 1.**
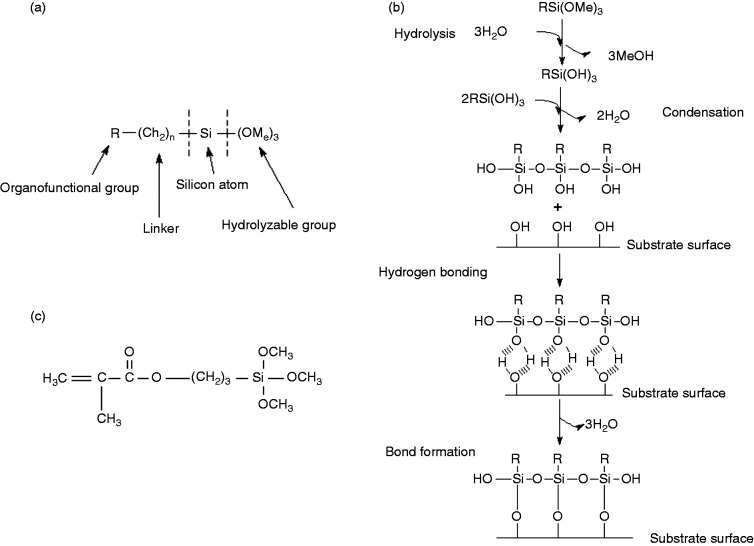
(a) General structure of silanes; (b) the hydrolytic deposition of silanes on substrates; and (c) the chemical structure of methacryloxypropyltrimethoxysilane (MPS).

Chemically modifying the surface of the radiopacifier may also prevent agglomerations and debonding from the PMMA matrix. For example, silane coupling agents (herein referred to as silanes) can be used to modify the wetting and adhesion characteristics of substrates and can create chemical bonds between organic and inorganic materials. Silanes are composed of an organofunctional group, a linker, a silicon atom and a hydrolyzable group (Figure 1(a)).^46^ The functional group (R group) mediates interactions with other materials and the length of the linker allows the functional group to extend farther from the inorganic substrate to improve particle dispersion characteristics.^47^ Silane functionalisation of surfaces occurs in four sequential steps (Figure 1(b)): (1) hydrolysis of the 3 labile groups; (2) condensation of the oligomers; (3) hydrogen bonding of the oligomers with hydroxyl groups on the substrate surface; and (4) covalent bonding with the substrate during drying/curing.^47^ The radiopacifiers previously discussed (TiO_2_ and ZrO_2_) both have hydrolytically stable surface oxides with sufficient hydroxyl functionality to allow coupling by the hydrolytic deposition process outlined.

The aim of the present study was to assess the suitability of anatase titanium dioxide (TiO_2_) and yttria-stabilised zirconium dioxide (ZrO_2_), as alternative radiopacifiers for PMMA bone cement, over conventional BaSO_4_ radiopacifier particles. The study also investigated whether silane treatment of these alternative radiopacifiers can further enhance *in vitro* mechanical, fatigue crack propagation and biological cement performance. Two commercial brands of cement, widely used in anchoring joint arthroplasties (Cemex Isoplastic and Palacos R), were also included in this study as reference materials for the clinically relevant properties studied.

## Materials and methods

### Materials

Anatase TiO_2_, yttria-stabilised ZrO_2_, methyl methacrylate with 75 ppm hydroquinone, N-N dimethyl-p-toluidine, benzoyl peroxide and methacryloxypropyltrimethoxysilane were purchased from Sigma Aldrich (Gillingham, UK). BaSO_4_ and Whatman grade 6 filters (185 mm diameter) were purchased from Fisher Scientific UK (Leicestershire, UK). Polymethyl methacrylate powder with a methylacrylate copolymer (Colacryl TS1713) was kindly provided by Lucite International UK (Southampton, UK). Cemex Isoplastic, which contains BaSO_4_ as a radiopacifier, was kindly provided by Tecres (Sommacampagna, Italy) and Palacos R, which contains ZrO_2_ that is not yttria-stabilised as a radiopacifier, was kindly provided by Heraeus (Newbury, UK). Alpha Minimum Essential Medium (α-MEM with nucleosides, L-glutamine, sodium pyruvate, lipoic acid, B12, biotin and ascorbic acid), foetal bovine serum and penicillin/streptomycin were purchased from Life Technologies Ltd (Paisley, UK).

### Particle size of radiopacifiers

The particle size distributions of BaSO_4_, anatase TiO_2_ and yttria-stabilised ZrO_2_ were examined using laser diffraction (Mastersizer X, Malvern Instruments Ltd, Worcestershire, UK) attached to an ultrasonic bath mixer. 1 g of the radiopacifier was added to 50 mL deionised distilled water in the ultrasonic bath mixer. This disperses the particles, preventing agglomerations in order to obtain accurate size readings. The suspension was measured using a 45 mm optical lens.

### Silane treatment

The silane coating procedure was based on industrial processes used to coat metallic surfaces and glassware.^46,48^ A 100 mL solution of 95%v/v ethanol was prepared and the pH adjusted to 4.5–5.5 using acetic acid. Methacryloxypropyltrimethoxysilane (MPS, also known as 3-(trimethoxysilyl)propyl methacrylate) was added at a 2%v/v concentration and allowed to hydrolyse at room temperature for 5 minutes. MPS was selected for this study as it has been previously used as an implant coating to enhance adherence to bone cements.^49,50^ This silane has three carbon atoms between the functional group and the silicon atom (Figure 1(c)). It is a gamma substituted silane, which is thermally stable up to 350 °C short-term and 160 °C long-term and will not be affected by the exothermic polymerisation reaction of bone cement and the temperatures experienced *in vivo*. MPS also has a similar reported toxicity to methyl methacrylate (LD50 > 3000 mg/kg).^46^ 5 g of radiopacifier powder (anatase TiO_2_ or yttria-stabilised ZrO_2_) was added to the solution and stirred for 24 hours at 25 °C. The suspension was allowed to settle and the upper clear solution decanted. The remaining slurry was filtered through a Whatman grade 6 filter. The slurry was rinsed twice with ethanol to remove excess unreacted silane before drying at 80 °C for 24 hours. Once dried, the powder was crushed using a pestle and mortar and passed through a 30-mesh sieve followed by a 300-mesh sieve to ensure a powder size of less than 50 μm.

### Fourier transform infrared spectroscopy of silane-treated radiopacifiers

To evaluate the success of the treatment process, Fourier Transform Infrared Spectroscopy (FT-IR) measurements were taken in transmission between 4000 cm^−1^ and 450 cm^−1^ with a resolution of 4 cm^−1^ using a Perkin Elmer Spectrum One FT-IR Spectrometer with FT-IR Spectrum software (Perkin Elmer, Massachusetts, USA). Potassium bromide (KBr) discs were prepared by combining 1 mg of the sample (MPS, TiO_2_, ZrO_2_ or silane-treated TiO_2_ and ZrO_2_) with 100 mg of potassium bromide (KBr) using a pestle and mortar until a fine uniform powder was obtained. The powder was then placed between two stainless steel disks and subjected to a force of 10 kN for 30 seconds, followed by 100 kN for a further 30 seconds to create discs. Two discs were prepared per sample and 30 scans per sample were performed to obtain an averaged spectrum.

### Cement preparation

The ISO5833:2002 (Implants for surgery - Acrylic resin cements) standard was used as a guideline for preparing cement samples, to ensure consistency between mixing batches.^51^ All the contents of the cement were equilibrated in air at 23 °C for 2 hours prior to mixing. Cement components were weighed and combined as outlined in Table 1. Samples were prepared containing 0, 5, 10, 15, 20 and 25%w/w of untreated TiO_2_ or ZrO_2_ and 10%w/w silane-treated TiO_2_ or ZrO_2_. Due to the differences in composition and manufacturing methods between the commercial and the formulated cements, samples containing 10%w/w BaSO_4_ were also prepared to establish the effect of the alternative radiopacifiers alone. Each powdered component was passed through a 35-mesh sieve (0.5 mm opening) before being weighed to an accuracy of ±0.01 g and blended together. Separately, each ingredient of the liquid component was weighed and mixed together. Both components were then introduced into a clean polypropylene bowl and hand-mixed at approximately 1 Hz with a polypropylene spatula for 1 minute. The mixture was then poured into a polytetrafluoroethylene (PTFE) mould to either polymerise for 24 hours or to measure the setting properties. Two commercially available bone cements (Cemex Isoplastic and Palacos R) were also mixed according to manufacturer’s instructions to act as reference materials. Following mixing and curing, surfaces were visually inspected and samples containing large pores (>0.5 mm) were discarded. Similarly, the results from samples with large pores in the fracture surfaces were discarded and additional samples tested.

**Table 1.** Composition of tested bone cements.

		Cemex isoplastic	Palacos R	Test samples					
Liquid	Total liquid (g)	13.3	18.8	16.9					
	Methyl methacrylate (%w/w)	99.1	98.0	98.5					
	N-N dimethyl-p-toluidine (%w/w)	0.9	2.0	1.5					
	Hydroquinone (ppm)	75.0	60.0	75.0					
Powder	Total powder (g)	40.0							
	Polymethyl methacrylate (%w/w)	84.3	84.5^a^	98.3	93.3	88.3	83.3	78.3	73.3
	Radiopacifier (%w/w)	13.0	15.0	0.0	5.0	10.0	15.0	20.0	25.0
	Benzoyl peroxide (%w/w)	2.7	0.5	1.7	1.7	1.7	1.70	1.7	1.7
Powder:liquid ratio		3.0	2.1	2.4					

^a^Poly(methyl methacrylate/methyl acrylate) powder.

### Radiopacity

To assess whether the alternative radiopacifiers provided similar radiopacity to BaSO_4_ containing cements and commercial products, radiographic images of 2 mm × 10 mm square cement samples were captured using a Kodak *in vivo* Imaging System FX Pro (Kodak Molecular Imaging™ Systems, USA) using an accelerating voltage of 35 kVp, 150 μA. The dimensions were selected to represent the minimum cement mantle thickness found *in vivo.*^52^ Images were acquired using Kodak Molecular Imaging™ V3 software and analysed using ImageJ (National Institutes of Health, Maryland, USA) to assess the optical density of samples in terms of average brightness of the image picture elements (pixels). The results were obtained as mean grey values, ranging from 0 (black) to 255 (white). A mean grey value of 255 would indicate complete absorption of X-rays, whilst a value of 0 would indicate complete transmission. Two samples for each test group were measured in air.

### Setting time and temperature

To ensure the setting time and temperature of the bone cements were not drastically altered by the alternative radiopacifiers, measurements and calculations for the setting properties were carried out according to the ISO5833:2002 standard using a high-density polyethylene mould and a K-type wire thermocouple connected to a Picotech ADC11 Data Logger (Cambridgeshire, UK).^51^ The ambient temperature was recorded prior to mixing and 25 g of cement was prepared as previously described. Temperature and time recording started when the liquid came into contact with the powder. Temperatures were recorded to an accuracy of ±0.5 °C. The setting temperature was calculated as the mean of the maximum setting temperature and the ambient temperature. The setting time was defined as the time when the setting temperature was reached.

### Surface energy

To investigate whether properties of the alternative radiopacifiers influenced the bulk hydrophilicity of the bone cement, surface energy measurements were performed according to the EN828 British Standards method.^53^ Small cylindrical bone cement samples (10 mm diameter, 2 mm thickness) containing 10%w/w BaSO_4_, TiO_2_, ZrO_2_, silane-treated TiO_2_ and silane-treated ZrO_2_ were prepared as previously described. The flat surfaces of the samples were polished with a 500-grit silicon carbide paper. Three test liquids were used in this experiment: water, methanol and glycerol. 2 μL of the test liquid was pipetted perpendicularly onto the surface of the sample and an image was taken of the profile of the droplet using a high definition digital camera. The images were then imported into ImageJ (National Institutes of Health, Maryland, USA) and the advancing and receding contact angles measured to give an average contact angle per sample (θ). Five samples were tested per group. A linear variation (y = mx + c) of Young’s equation was used to calculate the total surface free energy of each sample. The interfacial tension values for the test liquids and the average contact angles for each liquid on a sample were used to calculate the surface free energy as outlined in the EN828 British Standards.^53^

### Compressive and bending properties

Compression and bending properties were determined using a Zwick Roell ProLine table-top Z050/Z100 materials testing machine with TestXpert II software (Zwick Testing Machines Ltd., Herefordshire, UK) following the ISO5833 standard to investigate the effect of the alternative radiopacifiers on the mechanical performance of the cement.^51^ Cylindrical compression samples (6 mm diameter and 12 mm height) were tested at a cross-head speed of 20 mm/min. Rectangular bending samples (75 mm length, 10 mm width and 3.3 mm thickness) were tested at a cross-head speed of 5 mm/min in four-point bending. Five compression and five bending samples were tested to determine average compression and bending properties.

### Fracture toughness

Linear elastic fracture mechanics tests using single-edge notched three-point bending samples (35 mm length, 10 mm width and 3 mm thickness) were used to determine changes in the fracture toughness due to the different radiopacifiers. A sharp chevron notch (4.5 to 5.5 mm in length) was created through the centre of the sample using a surgical scalpel blade mounted onto a modified microtome, as described by Evans^23^ and the initial crack length and specimen dimensions measured using a travelling microscope (Pye Scientific, Cambridge, UK). The specimens were loaded at a crosshead speed of 5 mm/min in three-point bending, with the span between the rollers set to 40 mm, until failure occurred. The load and displacement were recorded and the critical stress intensity factor (K_ic_) of the cement was calculated as described in the ISO13586:2000 standard and in previous studies.^6,54,55^ Five samples were tested for each group to produce mean fracture toughness values.

### Fatigue crack growth rates

To accurately assess the influence of the alternative radiopacificers on cement crack propagation, fatigue crack growth rate tests were performed using disc-shaped compact tension specimens, with dimensions conforming to the ASTM E399 standard and as described by Ayre et al.^6,55,56^ A modified microtome was used to cut a chevron notch, which ensured symmetrical crack growth. The initial crack length and specimen dimensions were measured using a travelling microscope (Pye Scientific, Cambridge, UK). In order to apply the load symmetrically across the sample, loose plates with oversize holes were used, which allowed the loading pins to rotate freely. The crack length was monitored using Krak-gauges and a constant current supply and amplifier designed and built by Evans.^23^ Tests were performed in an Instron environmental chamber (Instron SFL, Bristol, UK) set at 37 °C and the current regulator and preamplifier were also kept in the environmental chamber to minimise drift. Two fatigue samples per group were tested until failure, using a 5 kN Dartec servohydraulic testing machine with an MTS FlexTest GT controller and MTS Multipurpose software (MTS, Eden Prairie, MN, USA).

Samples were cyclically loaded in load control with a sine wave at 5 Hz between 100 N and 10 N (R-ratio of 0.1), allowing for measurements of crack growth rates through stress intensities of 0.3 to 0.9 MPam^1/2^. Prior to loading, samples were subject to a pre-cracking procedure to ensure an initial steady crack growth rate of 10^−9^ m/cycle. For pre-cracking, a high cyclic load of 200 N at 5 Hz was applied and the load reduced by 10% after every 0.2 mm of crack growth. The crack length and number of cycles were measured during loading and the crack growth rate (da/dN in m/cycle) was calculated for every 0.2 mm of crack growth. The corresponding stress intensity factor range (ΔK) for each sample and crack length (ranging from 6–12 mm, in steps of 0.2 mm) was calculated based on the minimum and maximum load, sample dimensions and crack length as described by Ayre et al.^6,55^ For each specimen, da/dN was plotted against ΔK on logarithmic axes. The Paris Law (da/dn = A ΔK^m^) was fitted to the results, yielding values for A (crack growth rate at ΔK = 1 MPam^1/2^) and m (change in crack growth rate over the range of ΔK measured).

### Hydroxyapatite (HA) nucleation

HA nucleation was determined according to the ISO23317:2007 standard.^57^ 2 mm thick by 10 mm square bone cement samples were prepared containing 10%w/w BaSO_4_, 10%w/w TiO_2_, 10%w/w ZrO_2_, 10%w/w silane-treated TiO_2_ and 10%w/w silane-treated ZrO_2_. Two samples for each group and time point were used. Each sample was stored in 30 mL of simulated body fluid (SBF) based on a solution developed by Kokubo et al.^58^ The samples and SBF were stored in a V-bottom container at 37 °C for 1 hour, 1 week and 4 weeks. After each time point, the samples were removed and washed twice with distilled deionised water to remove any unattached precipitates. The samples were then desiccated without heat to prevent crystallisation. The sample surface was examined by X-ray diffraction (XRD) using a Philips PW3830 X-ray generator with a PW1710 Diffractometer control (Philips Research, Eindhoven, The Netherlands) and X’pert Industry v1.1c software (PANalytical B.V., Almelo, The Netherlands). The samples were scanned between 3° and 50° (2θ) at a scan speed of 0.020°/s. After XRD, samples were imaged using scanning electron microscopy (SEM) for visual inspection of hydroxyapatite crystals as described below.

### Scanning electron microscopy

The fatigue fracture surfaces and hydroxyapatite test samples were gold coated using an k550x sputter coater (Emitech,Kent,UK) and imaged using an EBT1 scanning electron microscope (SEM Tech Ltd, Suffolk, UK) at 15KeV.

### Cell attachment and viability

The MC3T3-E1 osteoblast precursor cell line (ATCC®, LGC Standards, Middlesex, UK) derived from mouse calvaria was used to investigate cell attachment and viability. Cells of passage 14–16 were maintained in α-MEM supplemented with 10% foetal bovine serum and 1% penicillin/streptomycin at 37 °C in 5% CO_2_. Four samples for each test group and time point were prepared (cylindrical 10 mm diameter by 2 mm height). The cylindrical samples were sterilized in 100% ethanol for 5 minutes and allowed to air dry under aseptic conditions. Samples were transferred to a 24 well-plate and approximately 6000 cells in 150 μL of supplemented culture medium was seeded on top of each sample and incubated for 4 hours at 37 °C in 5% CO_2_ to allow attachment. After 4 hours, each well was flooded with 750 μL of supplemented culture medium and incubated for 1, 4 and 24 hours. After each time point, the medium was removed and the samples washed with phosphate buffer saline (PBS) to remove non-adherent cells. Adherent cells were then fixed with 10%v/v neutral buffered formalin for 10 minutes before washing with PBS and staining with 1%w/v toluidine blue for 10 minutes. The stain was removed and the samples were washed twice with distilled water. The samples were left to dry and four random areas on the surface of each sample were imaged using an Olympus IX50 light microscope (Olympus, Southend-on-Sea, UK). The number of adherent cells were counted using ImageJ software (National Institutes of Health, Maryland, USA). The experiment was performed in triplicate.

For the cell viability assay, cement samples were prepared and sterilised as previously described. 3000 cells in 500 μL of supplemented culture media were seeded on each sample surface and the samples incubated at 37 °C in 5% CO_2_ for 3 and 7 days, with the medium replaced every 2 days. At each time point, the samples were washed with PBS and moved to a new 24-well plate. 500 μL of phenol-free α-MEM and 100 μL of CellTiter 96® AQueous One Solution Proliferation Assay (Promega Corporation, WI, USA) were added to each well and the plate was incubated in the dark for 2 hours. Wells containing cement samples without any cells were used as controls. After 2 hours, 100 μL of the assay medium was moved to a 96-well plate and the absorbance was measured at 490 nm using a Packard Spectracount plate reader (Buckinghamshire, UK). The control sample absorbance was subtracted from the absorbance of samples with cells to account for background generated by the cement. The experiment was performed in triplicate.

### Statistical analysis

Statistical analyses were performed using GraphPad Prism version 8 for Mac (GraphPad Software, La Jolla California USA). Groups of data were tested for normality using a Shapiro-Wilk test. For data that approximately followed a normal distribution, a parametric analysis of variance (ANOVA) was performed with a Tukey-Kramer post hoc analysis (compressive strength, bending strength, cell attachment, cell viability and surface energy). For data that deviated from a normal distribution, a non-parametric Kruskal-Wallis test was performed with a Dunn’s post hoc analysis (bending modulus and fracture toughness). The confidence interval for establishing significant differences between groups was set at 95% (p < 0.05).

## Results

### Radiopacifier particle size

**Figure 2.**
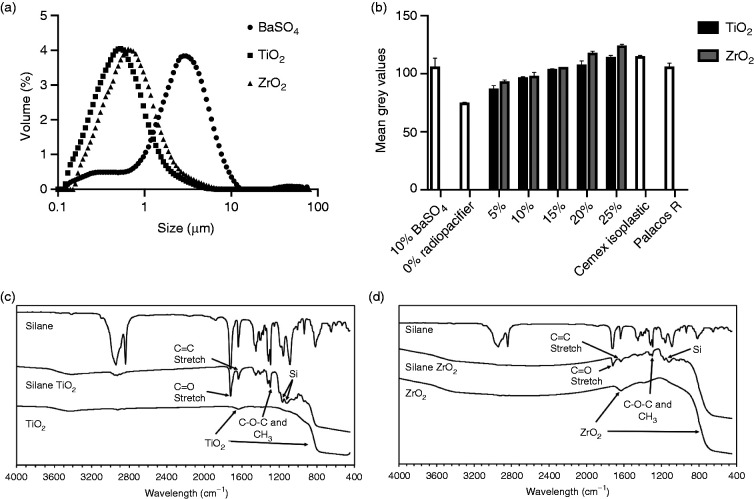
(a) TiO_2_ and ZrO_2_ radiopacifiers had sub-micron particle sizes, whilst BaSO_4_ particles were larger with a smaller volume percentage in the sub-micron region. (b) Analysis of X-ray images showed that greater than 10%w/w TiO_2_ or ZrO_2_ resulted in similar radiopacity to the 10%w/w BaSO_4_ cements. Fourier transform infrared spectroscopy confirmed the silane coating on (c) TiO_2_ and (d) ZrO_2_ radiopacifiers by the presence of organic and silicon peaks.

The volume median diameters (D_50_) for BaSO_4_, TiO_2_ and ZrO_2_ were 2.72 μm, 0.53 μm and 0.74 μm respectively (Figure 2(a)). Both TiO_2_ and ZrO_2_ had unimodal distributions in the sub-micron region whilst BaSO_4_ had a small volume percentage of sub-micron particles with most of the particles in the 1 to 10 μm region.

### Radiopacity

Approximately 10 to 20%w/w TiO_2_ or ZrO_2_ were required to achieve similar radiopacity to the 10%w/w BaSO_4_ samples and the commercial products (Figure 2(b)). Increasing the amount of TiO_2_ and ZrO_2_ in the cement samples resulted in a dose-dependent increase in radiopacity. At high concentrations (20 and 25%w/w), ZrO_2_ gave slightly higher mean grey values compared to TiO_2_. PMMA without a radiopacifier had the lowest radiopacity when compared to all other samples.

### Fourier transform infrared spectroscopy of silane-treated TiO_2_ and ZrO_2_

FT-IR spectra for silane-treated TiO_2_ and ZrO_2_ (Figure 2(c) and (d), respectively) confirmed that the particles were coated due to the presence of organic peaks similar to those of MPS. The most prominent peaks were the C = O stretch at 1730 cm^−1^, the C = C stretch at 1600 cm^−1^, the C-O-C and CH_3_ peaks around 1300 cm^−1^ and the two peaks at 1100 cm^−1^ and 1200 cm^−1^ associated with silicon. Both radiopacifiers showed characteristics of their original untreated material in the form of a small broad peak at around 1600 cm^−1^ and a reduction in intensity for wavelength values below 900 cm^−1^.

### Setting time and temperature

Cement samples containing ZrO_2_ had short setting times (Table 2, 10%w/w ZrO_2_, 10%w/w silane ZrO_2_ and Palacos R). 10%w/w silane ZrO_2_ and Cemex samples had the lowest maximum temperature, whilst 10%w/w TiO_2_ and 10%w/w ZrO_2_ had the highest maximum temperature. Silane treatment of both the TiO_2_ and ZrO_2_ slightly reduced the maximum temperature.

**Table 2.** Setting properties and average surface energy of commercial bone cements and cements containing various radiopacifiers.

Sample	Setting time (s)	Setting temperature (°C)	Maximum temperature (°C)	Surface energy (mN/m)
Cemex	738	46.5	69.8	
Palacos R	479	52.1	78.5	
0%w/w radiopacifier	615	51.4	81.6	30.6 ± 3.8
10%w/w BaSO_4_	665	49.2	76.2	28.8 ± 3.0
10%w/w TiO_2_	631	52.9	82.1	44.5 ± 3.5^+++^^/^***
10%w/w silane TiO_2_	579	51.0	79.6	41.7 ± 4.2^+++^^/^**
10%w/w ZrO_2_	441	53.3	82.2	36.7 ± 3.8
10%w/w silane ZrO_2_	442	46.1	69.1	41.6 ± 6.2^+++^^/^*

^+++^p < 0.001 compared to 10%w/w BaSO_4_; * p < 0.05, **p < 0.01, ***p < 0.001 compared to 0%w/w radiopacificer.

### Surface energy

Cements without a radiopacifier and cements with 10%w/w BaSO_4_ had a significantly lower surface energy compared to cements with TiO_2_ as their radiopacifier (Table 2, p < 0.01, untreated and silane-treated). The addition of both untreated and silane-treated TiO_2_ resulted in a more hydrophilic cement. There were no significant differences between the surface energies of TiO_2_ and ZrO_2_ containing samples (p > 0.05 for both untreated and silane-treated samples). Silane treating TiO_2_ and ZrO_2_ did not significantly alter the surface energy of the samples when compared to untreated TiO_2_ and ZrO_2_ (p > 0.05). Interestingly however, silane-treated ZrO_2_ samples had a significantly higher surface energy when compared to cements with no radiopacifier (p = 0.01) and cements with 10%w/w BaSO_4_ (p = 0.002) as the radiopacifier.

### Compressive and bending properties

**Figure 3.**
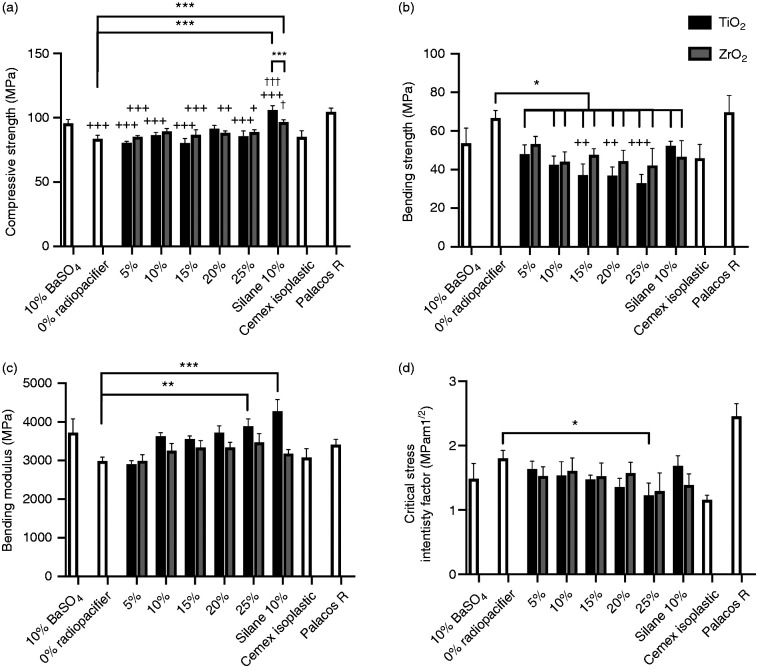
(a) Compressive strength, (b) bending strength, (c) bending modulus and (d) fracture toughness of commercial cements, cement samples containing 10%w/w BaSO_4_, 0 to 25%w/w TiO_2_ or ZrO_2_ and 10%w/w silane-treated TiO_2_ or ZrO_2_. Addition of radiopacifiers increased the bending modulus and decreased the bending strength and fracture toughness of samples in a dose-dependent manner. Silane-treating TiO_2_ improved the compressive strength, bending modulus and bending strength of the samples compared to untreated TiO_2_ samples. (*p < 0.05, ** p < 0.05, *** p < 0.001; + p < 0.05, ++ p < 0.01, +++ p < 0.001 compared to 10% BaSO_4_; † p < 0.05, †† p < 0.01, ††† p < 0.001 compared to 10%w/w radiopacifer).

Figure 3(a) shows the compressive strength of cement formulations with different radiopacifiers. PMMA without a radiopacifier had a significantly lower compressive strength when compared to PMMA with 10%w/w BaSO_4_ (p < 0.001). Overall, no trends were observed in compressive strength with the addition of up to 25%w/w TiO_2_ or ZrO_2_ to the cements. Cements containing 5, 15, 20 and 25%w/w untreated TiO_2_ (p < 0.001) and 5, 15, 20 and 25%w/w ZrO_2_ (p < 0.05) had significantly lower compressive strengths compared to 10%w/w BaSO_4_ cement. Silane treatment of TiO_2_ and ZrO_2_ significantly increased the compressive strength compared to the cement samples with the same amount of untreated radiopacifier (10%w/w TiO_2_ and 10%w/w ZrO_2_, p < 0.05). Silane-treated 10%w/w TiO_2_ had a significantly higher compressive strength compared to 10%w/w BaSO_4_ and cement without a radiopacifier (p < 0.001). Cements with silane-treated 10%w/w ZrO_2_ had a significantly higher compressive strength compared to cements with no radiopacifier (p < 0.001). All compressive strength values were similar to the two commercially available products (Cemex Isoplastic and Palacos R).

Addition of greater than or equal to 5%w/w TiO_2_ (p < 0.01) or 10%w/w ZrO_2_ (p < 0.001) to PMMA significantly decreased its bending strength (compared to 0%w/w radiopacifier, Figure 3(b)). Similarly, cement samples containing silane-treated TiO_2_ or silane-treated ZrO_2_ had a significantly lower bending strength compared to PMMA with no radiopacifier (p < 0.05). Increasing the amount of TiO_2_ in the samples caused a dose-dependent reduction in bending strength, with 15%w/w TiO_2_ or more having a significantly lower bending strength compared to 10%w/w BaSO_4_ (p < 0.01). This was not the case for ZrO_2_ samples, which although appeared to have a reduction in bending strength was not significantly different to 10%w/w BaSO_4_ (p > 0.05). There was no significant difference in bending strength between TiO_2_ and ZrO_2_ containing cements (p > 0.05) and no significant differences between 10%w/w untreated and silane-treated TiO_2_ or ZrO_2_. Palacos R had a significantly higher bending strength when compared to all cement samples (p < 0.01), except 0%w/w radiopacifier samples. Cemex Isoplastic was not significantly different to any formulation (p > 0.05), with the exception of 0%w/w radiopacifier samples, which had significantly higher bending strength (p < 0.001).

A trend was observed whereby increasing the amount of TiO_2_ and ZrO_2_ resulted in an increase in the bending modulus of the cements (Figure 3(c)). The bending moduli for 25%w/w TiO_2_ samples and silane-treated TiO_2_ samples were significantly higher than that of samples without a radiopacifier (p = 0.009 and p = 0.0008 respectively). No significant differences were observed between all formulations and 10%w/w BaSO_4_ samples. Although TiO_2_ containing samples appeared to have a higher modulus than ZrO_2_ containing samples at higher concentrations, this was not statistically significant, even for the silane-treated samples, where a large difference was observed (p > 0.05). All cement formulations had similar bending moduli to the commercial products, with the exception of silane TiO_2_, which had a significantly higher modulus than Cemex Isoplastic (p = 0.01).

Average compressive strength, bending strength and bending modulus values for the tested cement formulations are shown in the supplementary materials (Table S1) along with their respective standard deviations.

### Fracture toughness

Similar to the bending strength, addition of radiopacifiers to PMMA appeared to adversely affect the fracture toughness of PMMA in a dose-dependent manner (Figure 3(d)). Samples with 25%w/w TiO_2_ had significantly lower fracture toughness values than PMMA samples without any radiopacifier (p = 0.04). There were no significant differences in fracture toughness between samples containing 10%w/w BaSO_4_ and all other formulations (p > 0.05); and silane treatment of TiO_2_ and ZrO_2_ did not significantly improve the fracture toughness of the samples. There were no significant differences between samples containing TiO_2_ and ZrO_2_ (for both untreated and silane-treated samples, p > 0.05). Fracture toughness values for all formulations were found to fall within the range of those obtained for the two commercial products (Cemex Isoplastic and Palacos R). Interestingly, Cemex Isoplastic was found to have a significantly lower fracture toughness when compared to PMMA without a radiopacifier (p = 0.005) and when compared to Palacos R cement (p = 0.0002). Cement samples with 20 and 25%w/w TiO_2_ and 25%w/w ZrO_2_ had significantly lower fracture toughness values than Palacos R cement (p < 0.05). Average fracture toughness values along with their respective standard deviations for the cements tested are shown in the supplementary materials (Table S1).

### Fatigue crack growth rate

**Figure 4.**
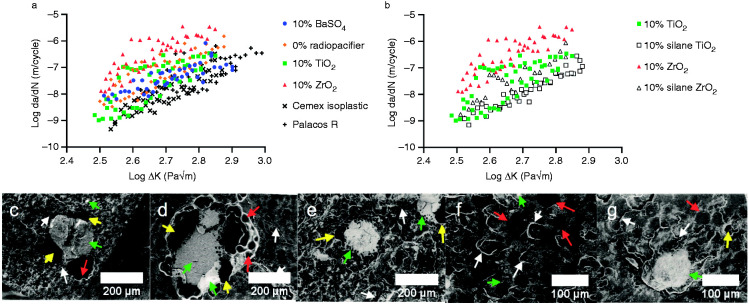
(a) Fatigue crack propagation results for commercial bone cement samples and samples containing no radiopacifier, 10%w/w BaSO_4_, 10%w/w TiO_2_ or 10%w/w ZrO_2_ as the radiopacifier. The addition of ZrO_2_ resulted in higher crack growth rates compared to other formulations. (b) Silane-treated TiO_2_ or ZrO_2_ reduced crack growth rates. Large radiopacifier agglomerates were observed on the fracture surfaces of (c) BaSO_4_, (d) TiO_2_ or (e) ZrO_2_ samples with voids surrounding the agglomerates. Silane treatment of (f) TiO_2_ and (g) ZrO_2_ reduced the size and number of agglomerates observed. Red arrows indicate PMMA beads, green arrows indicate radiopacifier agglomerations, yellow arrows indicate pores/voids and white arrows indicate microcracks.

10%w/w ZrO_2_ had the highest crack growth rates over the stress intensities tested (Figure 4(a)). 10%w/w TiO_2_ had similar crack growth rates compared to 10%w/w BaSO_4_ samples and radiopacifier-free samples. The commercial cements had the lowest crack growth rates. This is also shown in Table 3 where A (crack growth rate at ΔK = 1 MPam^1/2^) and m (change in crack growth rate over the range of ΔK measured) were both lower for the commercial cements. Silane treating TiO_2_ and ZrO_2_ resulted in a slight downward shift of the fatigue crack propagation plots, indicating improved crack growth resistance (Figure 4(b)). Fitting the Paris Law to the data showed a reduction in the crack growth rates at ΔK = 1 MPam^1/2^ (A coefficient), however no substantial change in crack growth rates over the range of ΔK tested (m coefficient, Table 3). At low stress intensities, silane-treated cements had lower crack growth rates, whilst at high stress intensities, comparable crack growth rates to the 10%w/w BaSO_4_ cement and the commercial cements were observed.

**Table 3.** Best-fit values of the Paris Law constants (A and m).

Sample	A	m	Correlation coefficient
Palacos R	3.73 × 10^−07^	4.65	0.61
Cemex	5.39 × 10^−07^	5.92	0.82
0% radiopacifier	3.53 × 10^−06^	5.49	0.69
10% BaSO_4_	4.83 × 10^−07^	3.73	0.67
10% TiO_2_	3.00 × 10^−06^	6.03	0.57
10% silane TiO_2_	6.80 × 10^−07^	5.94	0.92
10% ZrO_2_	6.92 × 10^−05^	6.88	0.73
10% silane ZrO_2_	5.51 × 10^−06^	6.76	0.84

A represents the crack growth rate (at ΔK = 1 MPam^1/2^) and m the change in crack growth rate over the range of ΔK measured.

Large radiopacifier agglomerations in the fracture surfaces were observed for the 10%w/w BaSO_4_, 10%w/w TiO_2_ and 10%w/w ZrO_2_ samples when viewed under SEM (Figure 4(c) to (e) respectively), with agglomerates ranging from 20 to 200 μm in length (up to one hundred fold the size of the individual radiopacifier particles). Large voids were also observed surrounding the agglomerations. Silane treatment improved the dispersion of TiO_2_ particles (Figure 4(f)), however for silane-treated 10%w/w ZrO_2_ large agglomerations were still present (Figure 4(g)). Both silane fracture surfaces however, appeared visibly rougher than the untreated TiO_2_ and ZrO_2_ fracture surfaces indicating a less brittle fracture (Figure 4(f) and (g)). Semi-circular microcracks were observed on the fracture surfaces of the silane-treated 10%w/w TiO_2_ samples, indicative of microcracks forming ahead of the crack tip (Figure 4(f)). Microcracks induce plastic deformation ahead of the crack tip and cause the main crack to deviate or branch out, slowing crack propagation and reducing the stress intensity at the tip of the crack.

### Hydroxyapatite nucleation

**Figure 5.**
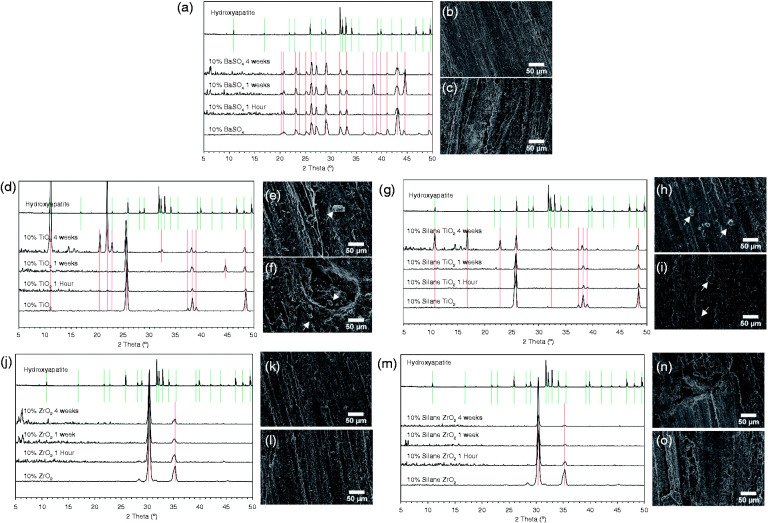
X-ray diffraction (XRD) spectra and scanning electron microscopy (SEM) images of (a to c) BaSO_4_, (d to f) TiO_2_, (g to i) silane-treated TiO_2_, (j to l) ZrO_2_ and (m to o) silane-treated ZrO_2_ samples following (b, e, h, k, n) 1 and (c, f, i, l, o) 4 weeks incubation in simulated body fluid. The nucleation of rod-like hydroxyapatite crystals was observed on cement samples containing TiO_2_ (untreated (e and f) and silane-treated (h to j)) as highlighted by alignment of XRD peaks with hydroxyapatite (d and g) and white arrows in the SEM images (e, f, h and i).

XRD detected the radiopacifiers on the surface of the cement samples, as shown by peaks at the same positions as the reference materials (radiopacifier powders, bottom spectra of Figure 5(a), (d), (g), (j), and (m)). XRD confirmed that the anatase phase of TiO_2_ and the tetragonal phase of ZrO_2_ were present (Figure 5(d) and (g) respectively). No HA was detected on the surface of cements containing BaSO_4_, ZrO_2_ or silane-treated ZrO_2_ for all time points (Figure 5(a), (j), and (m), respectively). This was confirmed with SEM images at 1 and 4 weeks, where no changes in surface topography or deposition of HA were observed (Figure 5(b), (c), (k), (l), (n), and (o)). For the BaSO_4_ samples, after 1 week in SBF an additional peak at 38.3° was observed (Figure 5(a)) however this peak did not align with those of HA and was not present at 4 weeks.

Cement samples containing TiO_2_ had peaks at approximately 11.0°, 22.0°, 22.8° and 32.3° after 4 weeks in SBF, which aligned with HA peaks (Figure 5(d)). Small crystal structures were observed on the surfaces of these samples under SEM after 1 week and 4 weeks incubation (Figure 5(e) and (f) respectively). Peaks at 44.5° after 1 week and 20.5° after 4 weeks were also detected, however these did not align with HA peaks.

Silane-treated TiO_2_ samples also had peaks at the same positions as HA after 4 weeks in SBF. These peaks were at approximately 11.0°, 16.9°, 22.8° and 32.3° (Figure 5(g)). Although several HA peaks were detected by XRD and observed using SEM, complete surface coverage with HA after 4 weeks was not observed.

### Cell attachment and viability

**Figure 6.**
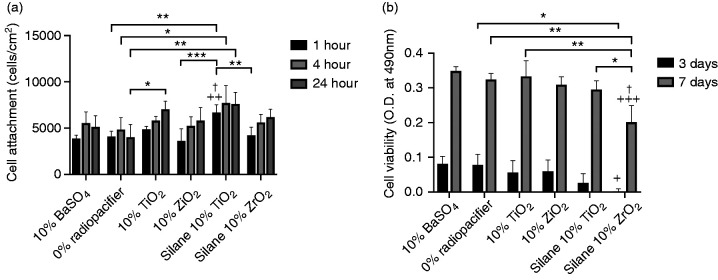
(a) Cell attachment to cement samples containing different radiopacifiers after incubation for 1, 4 and 24 hours; and (b) cell viability following incubation for 3 and 7 days. Samples containing silane-treated TiO_2_ had a significantly higher number of attached cells after 1 hour incubation compared to samples containing 10%w/w BaSO_4_. Silane-treated ZrO_2_ samples had a significantly reduced cell viability at 3 and 7 days compared to samples containing 10%w/w BaSO_4_. (* p < 0.05, ** p < 0.01, *** p < 0.001; + p < 0.05, ++ p < 0.01, +++ p < 0.001 compared to 10%w/w BaSO_4_; † p < 0.05 compared to 10%w/w radiopacifer).

Figure 6(a) shows the number of cells attached to the surface of the cement samples after 1, 4 and 24 hours incubation. After 1 hour incubation, samples containing silane-treated TiO_2_ had significantly higher numbers of attached MC3T3-E1 cells when compared to the 10%w/w BaSO_4_ (p = 0.001), 0%w/w radiopacifier (p = 0.002), 10%w/w TiO_2_ (p = 0.04), 10%w/w ZrO_2_ (p = 0.0004) and silane-treated ZrO_2_ (p = 0.004) cements. There were no other significant differences at 1 hour. At 4 and 24 hours, silane-treated TiO_2_ had significantly higher numbers of attached MC3T3-E1 cells when compared to 0%w/w radiopacifier cements (p = 0.03 for 4 hours and p = 0.004 for 24 hours). Interestingly at 24 hours, 10%w/w TiO_2_ cements also had significantly higher number of attached cells when compared to 0%w/w radiopacifier cements (p = 0.03).

Although MC3T3-E1 cells attached more readily on the surface of silane-treated TiO_2_ samples, the results from the cell viability assay showed that the samples with silane-treated TiO_2_ and ZrO_2_ had slightly lower 490 nm absorbance values, indicating a lower number of viable cells (Figure 6(b)). This reduction was only statistically significant for silane-treated ZrO_2_ samples, which at 3 days had significantly lower cell viability when compared to 10%w/w BaSO_4_ (p = 0.03) and 0%w/w radiopacifier (p = 0.04) samples. At 7 days this was also the case, with silane-treated ZrO_2_ samples having a significantly lower cell viability when compared to 10% BaSO_4_ (p = 0.0009), 0%w/w radiopacifier (p = 0.004), 10%w/w TiO_2_ (p = 0.003), 10%w/w ZrO_2_ (p = 0.01) and silane-treated TiO_2_ (p = 0.03) samples. Interestingly between days 3 and 7 there was still an increase in absorbance values for the silane-treated TiO_2_ and silane-treated ZrO_2_ samples, indicating the cells were still proliferating, however at a potentially lower rate than on other cement surfaces.

Table S2 (supplementary materials) shows the average cell attachment and cell viability values and standard deviations for the tested cements.

## Discussion

The addition of radiodense inorganic particles to PMMA allows the cement to be observed using X-ray imaging, however previous studies have shown that this approach can adversely influence the mechanical and fatigue crack propagation of the material as well as induce an osteolytic biological response, potentially contributing to cement failure. Substantial research has been undertaken to develop novel radiopacifiers, particularly based on nano-particles;^38,39^ however concerns regarding environmental and systemic toxicity have limited their clinical translation.^41^ This study has therefore investigated whether alternative radiopacifiers (anatase TiO_2_ or yttria-stabilised ZrO_2_) offer any physicochemical or biological advantages over conventional BaSO_4_ radiopacifiers and whether silane treatment can further enhance cement properties. To investigate this, a model cement was formulated and the conventional radiopacifier (BaSO_4_) substituted to investigate the impact of the alternative radiopacifier alone. Commercial products were included in this study as reference materials for the ISO/ASTM standard mechanical, fracture toughness and fatigue crack propagation tests.

While the laser diffraction results show that the TiO_2_ and ZrO_2_ powders used in this study were sub-micron, the particle diameters measured were not considered to be within the nano-scale according to ISO definitions (between 1 nm to 100 nm).^59^ For this reason, and due to the agglomerations observed, the differences in results between the different radiopacifiers are likely not attributed to size differences, but rather to the properties of the materials.

The ability of a material to attenuate and absorb X-rays is governed by the energy levels used by the imaging equipment and the atomic number and density of the material, which in turn affects what is known as its mass attenuation coefficient.^60^ Beyond energy levels of around 15 keV, PMMA does not attenuate X-rays effectively (as shown in Figure 1(b) and Fig. S1), highlighting the need for radiopacifiers. Addition of 10%w/w or more TiO_2_ or ZrO_2_ to PMMA gave similar radiopacity to 10%w/w BaSO_4_ and commercial cements. At higher concentrations (20 and 25%w/w), ZrO_2_ had slightly higher mean grey values when compared to TiO_2_. This aligns with the theoretical transmitted intensity for ZrO_2_ at 35 keV, which is substantially lower than that of TiO_2_ and BaSO_4_ (Table S3). Clinically however, X-ray irradiation is performed at higher energy levels (≈70 keV) to minimise radiation doses. At this level, the transmitted intensity for titanium is greater (Table S3) and may result in reduced radiopacity. Future work would therefore be required to establish the clinical performance of the alternative radiopacifiers, taking into account this higher energy levels.

FT-IR results confirmed that the silane was bound to the surface of the radiopacifiers, similar to other studies using the same silane (MPS) and similar protocols to effectively coat titanium dental implants and yttria-stabilised zirconium ceramics.^61,62^

The higher thermal conductivity of TiO_2_ did not drastically alter the setting properties and heat dissipation of bone cement. Higher levels of metal oxide fillers (≃40%w/w) may be required to observe such an effect as reported in the literature.^63–66^ Chou and Mariatti^67^ found the peak setting temperature of bone cement to reduce with increased nano-TiO_2_ loading. Interestingly the setting time was reduced when using 10%w/w yttria-stabilised ZrO_2_ as also observed with Palacos R, which uses a non-stabilised ZrO_2_ radiopacifier. It is speculated that the surface properties of ZrO_2_ may influence the orientation of polymer chains, which may influence the free radical polymerisation reaction.^68^

PMMA is hydrophobic in nature, as reflected by its low surface energy, and the addition of hydrophilic radiopacifiers resulted in an overall increase in surface energy. Reported surface energy values for commercial bone cements (CMW1 ≈ 29.4 mN/m),^69^ native PMMA (≈39 mN/m),^70^ BaSO_4_ (≈33 mN/m)^71^ and ZrO_2_ (≈42 mN/m)^72^ were consistent with those obtained experimentally for the modified PMMA samples (Table 2). Addition of 10%w/w TiO_2_ to PMMA significantly increased the surface energy of the material, likely due to the high surface energy of native anatase TiO_2_ (≈91 mN/m).^46^ The presence of a silane did not further significantly alter the surface energy of the cements, likely due to the negligible amounts of silane present in relation to the bulk of the cement.

No clear trend in compressive strength was observed when increasing TiO_2_ or ZrO_2_ concentrations up to 25%w/w. Fukuda et al.^73,74^ also showed no dose-dependent effect on compressive strength with the addition of up to 20%w/w rutile TiO_2_ to bone cement. Under the high compressive cross-head speeds used, the weaker PMMA beads or the interface between PMMA and the radiopacifier are likely to fail before the stiffer TiO_2_ or ZrO_2_ particles. Silane treatment of the TiO_2_ and ZrO_2_ however significantly increased the compressive strength. Goto et al.^75^ observed similar results whereby silane-treating nano-TiO_2_ (50%w/w) resulted in increased compressive strength. Coating the high surface energy (hydrophilic) radiopacifier with a low surface energy (hydrophobic) silane coupling agent, would improve the compatibility with PMMA and prevent agglomerates.^46^ Li et al.^76^ found coating TiO_2_ with a surfactant known as Span 60 increased the overall hydrophobicity of TiO_2_ and prevented it from agglomerating.

The proportional increase in bending modulus with increased radiopacifier content can be linked to stiffer particles replacing the softer PMMA particles. After silane treating the TiO_2_, a significant increase in bending modulus was observed, indicating enhanced integration with the PMMA matrix. The effect however was not observed for silane-treated ZrO_2_ samples. For TiO_2_ samples, the increased modulus resulted in a dose-dependent decrease in bending strength. Chou and Mariatti^67^ showed similar results where addition of nano-TiO_2_ to bone cement decreased the strain at break and flexural strength, again linked to increased agglomerates and void content. Fukuda et al.^73,74^ also found the same trend where bending modulus increased between 5 and 20%w/w rutile TiO_2_, causing a dose-dependent decrease in bending strength. The bending modulus and strength of bone cement containing ZrO_2_ however appeared to be less concentration dependent. Silane treatment of TiO_2_ reversed the detrimental effect on the bending strength; which is attributed to enhanced dispersion and adhesion to the PMMA matrix. Poor adhesion of the radiopacifier would result in detachment from the PMMA matrix under strain, creating voids and weakening the cement. This hypothesis is further reinforced by the fact that plain PMMA (with no radiopacifier) had the highest bending strength values, highlighting the role radiopacifiers play in initiating cement failure. The fracture toughness results reflected those of the bending strength, with a dose dependent decrease with increasing TiO_2_ concentrations. Similar to the bending strength, the fracture toughness of ZrO_2_ samples were less affected by the amount of radiopacifier incorporated into the cement. Interestingly however silane treatment of TiO_2_ and ZrO_2_ did not significantly enhance the fracture toughness of the samples. This may be due to the high crack propagation rates associated with this type of test. Further fatigue testing was performed to establish differences under low crack propagation rates.

The fatigue crack propagation tests showed that the radiopacifier-free samples did not have the lowest crack growth rates. This supports the argument put forward by several authors that radiopacifiers can also reduce crack propagation rates.^24–31^ Compared to the 10%w/w BaSO_4_ and the commercial cements, the addition of 10%w/w TiO_2_ or ZrO_2_ increased fatigue crack growth rates. Closer examination of the fracture surfaces identified large agglomerates for all the radiopacifiers tested with visible voids surrounding them. These features may act as stress concentrations under load and sites for crack initiation.^15–20,32,77^ Yttria-stabilised ZrO_2_ was expected to reduce fatigue crack propagation in the cement due to its transformation toughening mechanism, however in this study it increased crack growth rates. When silane treating TiO_2_ and ZrO_2_, an improvement in the crack growth rates were observed. SEM images of the fracture surfaces of the silane-treated TiO_2_ and ZrO_2_ samples showed fewer agglomerations and rougher fracture surfaces. Better dispersion would create fewer stress concentrations (known to initiate cracks) and enhanced adhesion would require higher stresses to de-bond the particle from the polymer matrix. As rigid particles do not deform easily, enhanced adhesion might also induce higher levels of plastic deformation in the surrounding polymer matrix, resulting in a tougher material. When functionalising nano-ZrO_2_ and nano-BaSO_4_ with a silane coupling agent, Gillani et al.^78^ found that cements with unfunctionalised radiopacifiers caused brittle failure whilst functionalised radiopacifiers caused plastic failure modes.

After incubation for 4 weeks in simulated body fluid, only the TiO_2_-containing samples showed evidence of HA nucleation. Studies have shown the negatively charged hydroxide and Lewis Base surface tension parameter of anatase TiO_2_ (used in this study) is more effective at inducing HA nucleation.^44,79^ The similarity between the surface energy of TiO_2_ (≈91 mN/m)^46^ and that of HA (≈72–95 mN/m)^80^ and the close lattice match with HA may also be a contributing factor.

MC3T3-E1, a murine calvarial osteoblast precursor cell line, was selected for this study as it is a well-documented model for studying attachment, proliferation and mineralisation on the surface of materials.^81^ The use of TiO_2_ and in particular, silane-treated TiO_2_, was found to enhance MC3T3-E1 cell attachment. This increased cell attachment may be attributed to the increased hydrophilicity of the TiO_2_ cements as previously discussed or due to the calcium ion interaction that occurs with TiO_2_, which selectively adsorbs serum proteins.^43^ Research by Wei et al.^82^ using a thin film of hexamethyldisiloxane (HMDSO) with and without oxygen plasma treatment showed hydrophilic surfaces preferentially bound fibronectin, which in turn correlated with increased MC3T3-E1 attachment, whilst hydrophobic surfaces preferentially bound albumin resulting in lower cell attachment. Fibronectin and vitronectin are often reported to play a vital role in osteoblast attachment and the improved rate of attachment between the bone cement samples may be linked to these serum proteins. A more rapid rate of osteoblast-like cell attachment as observed in this study has the potential to enhance the rate of osseointegration of bone cement. Fukuda et al.^73^ demonstrated this in an *in vivo* rabbit femoral model where addition of rutile TiO_2_ to bone cement was found to enhance osteoconductivity. Due to the higher acidity, lower surface zeta potential and closer lattice match of anatase with HA when compared to rutile,^44^ there is potential to achieve further osteogenic benefits when using anatase form of TiO_2_ as a radiopacifier.

Although silane-treated TiO_2_ samples had increased MC3T3-E1 cell attachment, longer-term incubation (3 and 7 days) showed slightly lower cell viability on the silane-treated TiO_2_ surfaces and significantly lower cell viability on the ZrO_2_ surfaces. A study investigating the toxicity of a range of silanes (including MPS) used to treat HA particles found that extracts from HA-treated particles only caused mild cytotoxicity.^83^ Other studies investigating the toxicity of silane-treated radiopacifiers in bone cement did not observe any *in vitro* or *in vivo* toxicity.^75,78,84^ It is probable that the silane itself is not overtly toxic, but may influence cell numbers through other mechanisms such as reduced cell metabolism or proliferation. This is supported by data between days 3 and 7 showing an increase in cell viability for the silane-treated TiO_2_ and silane-treated ZrO_2_ samples, however at lower proliferation rates than on other cement surfaces. Future research should explore the effect of the silane (MPS) on the expression of cell proliferation markers, such as Ki67, proliferating cell nuclear antigen (PCNA) and minichromosome maintenance 2 (MCM-2).

## Conclusion

This study has investigated the potential for using anatase TiO_2_ and yttria-stabilised ZrO_2_ as alternative radiopacifiers for PMMA bone cement. Addition of greater than 10%w/w TiO_2_ and ZrO_2_ resulted in a similar radiopacity to 10%w/w BaSO_4_ and commercial products when tested at 35 keV. The addition of anatase TiO_2_ and yttria-stabilised ZrO_2_ to bone cement however resulted in a reduction in bending strength and fracture toughness, which was dose dependent for TiO_2_; this reduction was reversed with silane treatment to the levels of the cements containing 10%w/w BaSO_4_ and the commercial products. These radiopacifiers were also found to increase the fatigue crack growth rates of the cement due to the formation of agglomerations and voids; this was again largely reversed by silane treatment of TiO_2_ with better management of crack propagation. Silane treating TiO_2_ in particular, enhanced radiopacifier dispersion and adhesion to the PMMA matrix. Furthermore, silane-treated TiO_2_ as a radiopacifer enhanced nucleation of HA and increased MC3T3-E1 cell attachment to the cement *in vitro,* with limited effect on cell viability. This work demonstrates some potential benefits associated with silane-treated anatase TiO_2_ as a radiopacifier for bone cement over conventional radiopacifiers.

## Supplemental Material

sj-pdf-1-jba-10.1177_0885328220983797 - Supplemental material for Alternative radiopacifiers for polymethyl methacrylate bone cements: Silane-treated anatase titanium dioxide and yttria-stabilised zirconium dioxide Supplemental material, sj-pdf-1-jba-10.1177_0885328220983797 for Alternative radiopacifiers for polymethyl methacrylate bone cements: Silane-treated anatase titanium dioxide and yttria-stabilised zirconium dioxide by Wayne Nishio Ayre Nicole Scully, Carole Elford, Bronwen AJ Evans, Wendy Rowe, Jeff Rowlands, Ravi Mitha, Paul Malpas, Panagiota Manti, Cathy Holt, Rhidian Morgan-Jones, James C Birchall, Stephen P Denyer and Sam L Evans in Journal of Biomaterials Applications
