# Supplementary material for: Alternative radiopacifiers for polymethyl methacrylate bone cements: Silane-treated anatase titanium dioxide and yttria-stabilised zirconium dioxide
Source: J Biomater Appl. 2021 Feb 11;35(10):1235–52. doi: 10.1177/0885328220983797 (PMC8058833; doi:10.1177/0885328220983797)
Supplement: sj-pdf-1-jba-10.1177_0885328220983797 - Supplemental material for Alternative radiopacifiers for polymethyl methacrylate bone cements: Silane-treated anatase titanium dioxide and yttria-stabilised zirconium dioxide [file sj-pdf-1-jba-10.1177_0885328220983797.pdf]

Supplementary materials

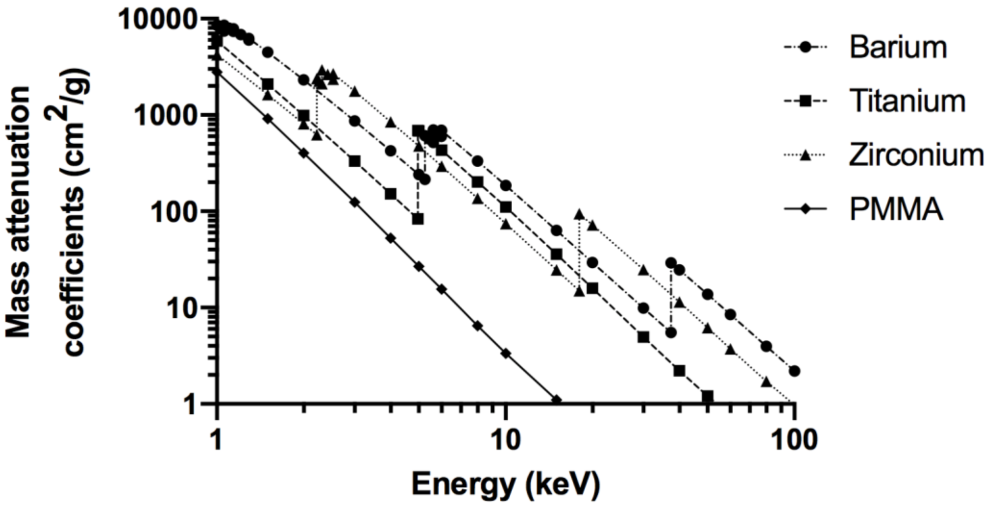

Fig. S1 Mass attenuation coefficients spectra for barium, titanium, zirconium and PMMA

Table S1 Average compressive strength, bending strength, bending modulus and fracture toughness values for tested cement compositions with standard deviations.

Sample	Compressive strength (MPa)	Bending strength (MPa)	Bending Modulus (MPa)	Critical stress intensity factor ($MPam^{1/2}$)
**10%w/w $BaSO_4$**	95.5 ± 2.7	53.8 ± 7.0	3725.5 ± 318.7	1.5 ± 0.3
**0%w/w radiopacifier**	83.7 ± 2.4^{+++}	66.8 ± 3.6	2991.5 ± 86.5	1.9 ± 0.2
**5%w/w TiO_2**	80.6 ± 1.0^{+++}	48.1 ± 4.3**	2906.2 ± 78.5	1.7 ± 0.2
**10%w/w TiO_2**	86.4 ± 1.9^{+++}	48.1 ± 4.3***	3637.2 ± 77.8	1.6 ± 0.2
**15%w/w TiO_2**	80.5 ± 3.1^{+++}	37.3 ± 5.2$^{++/***}$	3559.9 ± 72.8	1.5 ± 0.1
**20%w/w TiO_2**	91.5 ± 2.3**	37.0 ± 4.0$^{++/***}$	3727.5 ± 156.3	1.4 ± 0.2
**25% w/w TiO_2**	85.6 ± 3.6^{+++}	33.0 ± 4.1$^{+++/***}$	3894.5 ± 166.7**	1.3 ± 0.2*
**5%w/w ZrO_2**	85.3 ± 0.8^{+++}	41.7 ± 5.2	2993.0 ± 137.3	1.6 ± 0.2
**10%w/w ZrO_2**	89.3 ± 2.0	41.7 ± 5.2***	3256.3 ± 167.4	1.7 ± 0.2
**15%w/w ZrO_2**	86.7 ± 3.4^{+++}	44.2 ± 4.6***	3341.5 ± 158.4	1.6 ± 0.2
**20%w/w ZrO_2**	88.2 ± 1.4^{++}	47.7 ± 2.9***	3343.8 ± 116.9	1.6 ± 0.2
**25% w/w ZrO_2**	88.8 ± 1.6^{+}	42.2 ± 8.0***	3471.8 ± 200.8	1.3 ± 0.3
**10%w/w Silane TiO_2**	106.0 ± 3.0$^{+++/***/†††}$	42.2 ± 8.0*	4338.0 ± 274.8***	1.7 ± 0.2
**10%w/w Silane ZrO_2**	96.5 ± 1.6$^{***/†}$	53.2 ± 1.6***	3181.8 ± 91.2	1.4 ± 0.2
**Cemex**	85.2 ± 4.2^{+++}	69.8 ± 7.8***	3082.8 ± 204.6	1.2 ± 0.1**
**Palacos R**	104.6 ± 2.9$^{+++/***}$	46.0 ± 6.5^{++}	3414.0 ± 123.2	2.5 ± 0.2

(++ $p<0.05$, +++ $p<0.001$ compared to 10% $BaSO_4$; * $p<0.05$, ** $p<0.01$, ***$p<0.001$ compared 0%w/w radiopacifer; † $p<0.05$, ††† $p<0.001$ compared to 10%w/w radiopacifer)

Table S2 Average MC3T3 cell attachment and viability results with standard deviations for tested cement compositions.

Sample	Attached cell density (cells/cm^2) 1 hour	4 hours	24 hours	Absorbance (OD at 490nm) 3 days	7 days
**10%w/w $BaSO_4$**	4125 ± 44	5550 ± 1060	5150 ± 1031	0.082 ± 0.018	0.349 ± 0.01
**0%w/w radiopacifier**	4100 ± 495	4850 ± 1106	4025 ± 1187	0.079 ± 0.025	0.325 ± 0.014
**10%w/w TiO_2**	4875 ± 287	5825 ± 409	7050 ± 727*	0.057 ± 0.028	0.334 ± 0.037
**10%w/w ZrO_2**	3625 ± 1130	5250 ± 856	5825 ± 1228	0.027 ± 0.022	0.296 ± 0.021
**10%w/w Silane TiO_2**	6700 ± 732$^{++/**/†}$	7725 ± 1604*	7625 ± 1064**	0.06 ± 0.027	0.31 ± 0.019
**10%w/w Silane ZrO_2**	4225 ± 767	5625 ± 750	6200 ± 729	0.003 ± 0.006$^{+/*}$	0.202 ± 0.039$^{+++/**/†}$

(+ $p<0.05$, ++ $p<0.01$, +++ $p<0.001$ compared to 10% $BaSO_4$; * $p<0.05$, ** $p<0.01$ compared 0%w/w radiopacifer; † $p<0.05$ compared to 10%w/w radiopacifer)

Table S3 Transmitted intensity values at 35 and 70keV

Material	Original Intensity, I_o (keV)	Density, ρ (g cm^{-1})	Mass attenuation coefficient, μ/ρ (cm^2/g)	Linear attenuation coefficient, μ (cm^{-1})	Material thickness, e (cm)	Transmitted Intensity, I (keV)
**Barium**	70	3.5	5.1	17.9	1	1.3 x 10^{-06}
	35		6.9	24.4	1	9.2 x 10^{-10}
**Titanium**	70	4.5	0.5	2.3	1	7.9
	35		3.6	16.2	1	3.3x10^{-06}
**Zirconium**	70	6.5	2.2	14.5	1	3.6x10^{-05}
	35		18.1	118.0	1	2.0x10^{-50}
**PMMA**	70	1.18	0.2	0.2	1	60.7
	35		0.3	0.3	1	25.5
